# Supplementary material for: Discovery of processive catalysis by an exo-hydrolase with a pocket-shaped active site
Source: Nat Commun. 2019 May 20;10:2222. doi: 10.1038/s41467-019-09691-z (PMC6527550; doi:10.1038/s41467-019-09691-z)
Supplement: Supplementary file 1 — Supplementary Information [file 41467_2019_9691_MOESM1_ESM.pdf]

Supplementary Information

Discovery of processive catalysis by an exo-hydrolase with a pocket-shaped active site

Victor A. Streltsov[1,15], Sukanya Luang[2,15], Alys Peisley[1], Joseph N. Varghese[1], James R. Ketudat Cairns[3], Sebastien Fort[4], Marcel Hijnen[5], Igor Tvaroška[6], Ana Ardá[7], Jesús Jiménez-Barbero[7], Mercedes Alfonso-Prieto[8], Carme Rovira[8,9], Fernanda Mendoza[10,11], Laura Tiessler-Sala[11], José-Emilio Sánchez-Aparicio[11], Jaime Rodríguez-Guerra[11,12], José M. Lluch[11,13], Jean-Didier Maréchal[11], Laura Masgrau[11,13], and Maria Hrmova[2,14]

[1]Commonwealth Scientific and Industrial Research Organisation, Materials Science and Engineering, Parkville Victoria 3052, Australia. [2]School of Agriculture, Food and Wine, University of Adelaide, Waite Campus, Glen Osmond South Australia 5064, Australia. [3]School of Chemistry and Center for Biomolecular Structure, Function and Application, Suranaree University of Technology, 30000 Nakhon Ratchasima, Thailand. [4]University Grenoble Alpes, Centre de Recherches sur les Macromolécules Végétales, 38041 Grenoble cedex 9, France. [5]GE Healthcare Life Sciences, Paramatta NSW 2150, Australia. [6]Institute of Chemistry, Slovak Academy of Sciences, 84538 Bratislava, Slovak Republic. [7]Centre for Cooperative Research in Biosciences, 48160 Derio-Bizkaia, Spain. [8]Departament de Química Inorgànica i Orgànica, Universitat de Barcelona, 08028 Barcelona, Spain. [9]Institució Catalana de Recerca i Estudis Avançats, 08010 Barcelona, Spain. [10]Departamento de Ciencias Químicas, Universidad Andres Bello, Sede Concepción, 4260000 Talcahuano, Chile.[11]Departament de Química, Universitat Autònoma de Barcelona, 08193 Bellaterra, Spain.[12]Institute of Chemical Research of Catalonia, The Barcelona Institute of Science and Technology, 43007 Tarragona, Spain. [13]Institut de Biotecnologia i de Biomedicina, Universitat Autònoma de Barcelona, 08193 Bellaterra, Spain. [14]School of Life Sciences, Huaiyin Normal University, 223300 Huaian, China.

[15]These authors contributed equally: Victor A. Streltsov and Sukanya Luang.

Correspondence and requests for materials should be addressed to M.H. (email: maria.hrmova@adelaide.edu.au)

Supplementary Note 1

In-solution NMR spectroscopy of thiocellobiose binding to recombinant HvExoI. To confirm our observations of Glc binding (Fig. 4b; top panel), thiocellobiose that is not hydrolysed by HvExoI[1], was added in an excess relative to the enzyme. Here, clear negative NOEs between specific thiocellobiose proton pairs were observed (Supplementary Fig. 4). A NOESY spectrum of free thiocellobiose was also acquired under the same conditions, which showed the presence of COSY-like cross-peaks that arose from zero quantum coherences (ZQCs), and not from dipolar interactions (NOE). This implied that free thiocellobiose tumbled with a rotational correlation time corresponding to a zero value for NOE. Thus, negative NOEs observed for thiocellobiose bound to HvExoI that were assigned as trNOEs, arose exclusively from bound thiocellobiose. Specifically, strong trNOEs of the anomeric proton of the non-reducing Glc (H-1´) with the intra-residual H-3´ and H-5´ were observed (Fig. 4b; bottom panel), indicating that the non-reducing glucopyranose ring of thiocellobiose was bound in the $^4C_1$ conformation. In fact, the β-anomeric proton of thiocellobiose at the reducing-end (H1β) also showed strong intra-residual trNOEs to H3 and H5, as expected for the $^4C_1$ conformation (Fig. 4b; bottom panel). The comparisons of NOEs between free and bound thiocellobiose (Supplementary Fig. 4) suggested that HvExoI adopted a conformational selection process for a thioglycosidic linkage. While in a free form, thiocellobiose exists in an ensemble of three diverse conformers, as deduced from the observation of the exclusive inter-residual NOEs H1´-H4 (syn), H1´-H-3 (anti-Psi) and H2´-H4 (anti-Phi)[2], in the trNOESY spectrum, the only strong trNOEs were those of inter-residual H´1-H4. Consequently, the data indicate that HvExoI selects a syn conformer from the available conformational distribution of thiocellobiose conformers.

Supplementary Note 2

G6SG-OMe binds across the active site of recombinant HvExoI. The reducing-end Glc moiety of G6SG-OMe aligned with the indole ring of Trp286 and Trp434 such that the intra-ring oxygen of the reducing-end Glc moiety (hydrophilic face) faced Trp286, while that of the non-reducing-end moiety (hydrophobic face) pointed to Trp434. The loop carrying Glu491 to Asn498 adopted a new position, compared to that in ligand-free recombinant HvExoI. This productive binding of G6SG-OMe was emphasised by the presence of the hydrogen bond (H-bond) of 2.7 Å formed between C2-OH of non-reducing Glc and Oδ2 of Asp285 (catalytic nucleophile), and by the water-mediated contact formed between Oε2 of Glu491 (acid/base) and S1 of G6SG-OMe.

Supplementary Note 3

Reciprocal docking of β-D-glucopyranosyl-(1,2)-D-glucose (G2OG) or Glc to obtain ternary HvExoI:Glc:G2OG complexes. G2OG or Glc was docked in the HvExoI:Glc complex to obtain ternary HvExoI:Glc:G2O complexes 1 and 2, which we considered to be structural intermediates during the Glc displacement route. To obtain complex 1, Glc was docked in the -1 subsite of the HvExoI:G2OG complex, derived from the crystal structure of HvExoI in complex with G2SG-OMe (PDB 6MD6). The lowest-energy solution (score of 65) was used as a starting point for 100 ns of the MD simulation (Supplementary Fig. 5). In this complex (Supplementary Fig. 6; left-top panel), G2OG after 50 ns abandoned its original binding position and bound with the non-reducing-end in the +1 subsite, while the sidechain of Tyr253, that in the HvExoI:G2SG-OMe complex (PDB 6MD6) forms the H-bond with the carbonyl O-atom of the Trp286 backbone, rotated to expose a large cavity next to the -1 subsite (Supplementary Fig. 6; left-bottom panel). To obtain complex 2, docking of G2OG was performed on the HvExoI:Glc complex (PDB 3WLH). In the docking solutions, G2OG bound with the non-reducing glucosyl moiety in the +1 subsite, while the reducing moiety remained solvent exposed at the putative +2 subsite (score 58) (Supplementary Fig. 6; right-top panel). In this stable complex after 90 ns of MD simulations (Supplementary Fig. 5), Tyr253 changed its conformation, although to the other side compared to that in complex 1, making the exposed lateral cavity next to the -1 subsite shallower (Supplementary Fig. 6; right-bottom panel).

Supplementary Note 4

Exploration of alternative binding sites by MD simulations and docking. We reasoned that incoming substrates could potentially bind to different sites than those at the +1 and putative +2 subsites, described in complexes 1 and 2 above, and to trigger Glc displacement form the -1 subsite through the +1 subsite, which could only then be occupied by a new substrate. To investigate this possibility, MD simulations of binary HvExoI:Glc complexes revealed that Glc stayed bound in the active site for the entire 200 ns simulation time (Supplementary Fig. 5), while Trp434 changed its conformation that affected the space available in the +1 subsite (Supplementary Fig. 7b, d). Most of the time (>75%) Trp434 tilted perpendicularly with respect to Trp286 that barely moved along simulations, and partially blocked the space assigned to Glc' (PDB 3WLH), or to Glc2 in recombinant HvExoI perfused with Glc (PDB 3WLO) (Supplementary Fig. 7b, d; Fig. 3). The CA-CB-CG-CD1 dihedral angle of Trp434 at approximately -50° indicated the closed conformation of Trp434, which could establish various interactions. One of most stable ones was the H-bond between HE2 of Trp434 and O6 of Glc, formed 15 ns from the simulation onset that was retained for 100 ns. The second, a minor conformation observed for Trp434 with the CA-CB-CG-CD1 dihedral angle of

around 50° (Supplementary Fig. 7c, e), corresponded to that seen in the crystal structure (PDB 3WLH), with the indole ring parallel to that of Trp286 and HE2 of Trp434 interacting with the catalytic acid/base Glu491 (Supplementary Fig. 7c, e). Next, docking of G2OG and β-D-glucopyranosyl-(1,3)-D-glucose (G3OG) (Supplementary Fig. 6, 8, complexes 3-6) performed on the MD simulation snapshot described above (Supplementary Fig. 7b, d) aimed to shed light on identifying substrate binding sites other than those of the +1 and putative +2 subsites. These docking solutions described below, identified a solvent-exposed lateral cavity for potential substrate binding (Supplementary Fig. 6, 8).

Complex 3: This complex presents an open and the most frequent solvent exposed conformation of Trp434, providing this residue was set to be flexible during docking calculations. G3OG bound (score 68) with its non-reducing-end in the lateral cavity and the reducing-end in the +1 subsite (Supplementary Fig. 8; left-top panel). However, within first 4 ns of the MD simulation, G3OG positioned its non-reducing-end in the +1 subsite and interacted with solvent exposed Trp434. After around 40 ns, Trp434 changed its conformation to that observed in the native structure with trapped Glc (PDB 3WLH), while G3OG bound with its non-reducing-end in the +1 subsite and the reducing-end solvent exposed, as seen in the structure with G2SG-OMe (PDB 6MD6) (Supplementary Fig. 8; left-middle panel). After around 2 ns, Tyr253 changed its conformation to that observed in complex 1. These re-arrangements remained stable up to 125 ns of the simulation (Supplementary Fig. 8; left-bottom panel).

Complex 4: As Trp434 was set to be rigid, the docking solution found Trp434 occupying the +1 subsite and G2OG bound (score 67) in the lateral cavity (Supplementary Fig. 8; right-top panel). However, during equilibration G2OG abandoned this lateral cavity and remained bound at the protein surface near Trp434, which blocked the +1 subsite, and for 100 ns interacted with Glu36 (Supplementary Fig. 8; right-middle panel). After 104 ns Trp434 changed its conformation to that observed in crystal structures, while G2OG entered the +1 subsite with its non-reducing-end first adopting a binding mode analogous to that observed in the crystal structure with G2SG-OMe (PDB 6MD6), and the Tyr253 sidechain rotated as seen in complexes 1 and 3. This system remained stable up to 150 ns of the simulation run (Supplementary Fig. 8; right-bottom panel).

Complex 5: The Trp434 sidechain presents an open and solvent exposed conformation. This docking solution (score 64) was selected as G2OG bound next to the lateral cavity, thus leaving the +1 subsite unoccupied, so that it could be used as the potential displacement route. However, this was not a stable binding mode of G2OG, which during the first 10 ns of simulation was translated into the +1 subsite and subsequently rapidly moved into the solvent (Supplementary Fig. 5). Trp434

ended up blocking the +1 subsite, as it was seen during HvExoI:Glc simulations (Supplementary Fig. 7b, d).

Complex 6: Although Trp434 could change its conformation in this complex, which is one of the lowest energy solutions (score 75), Trp434 partially blocked the +1 subsite. G3OG bound initially to the lateral cavity, however further progression of MD simulations revealed that this was not a stable binding mode, as G3OG moved away in less than 10 ns (Supplementary Fig. 5). Trp434 remained blocking the +1 subsite for the next 40 ns of the MD simulation run.

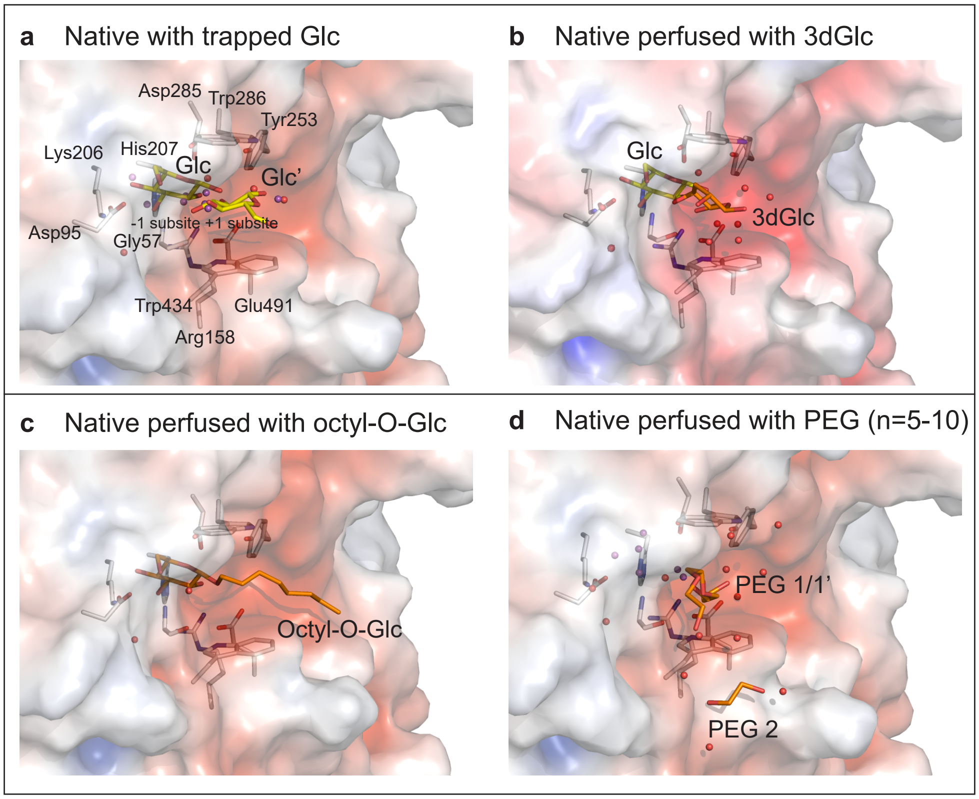

Supplementary Figure 1. Native HvExoI with trapped Glc, and with perfused 3dGlc, octyl-O-Glc or PEG (n=5-10).

a, Native HvExoI with trapped Glc. The Glc molecule (Glc and Glc', carbons: yellow sticks) at 0.5 occupancy oscillates between the -1 and +1 subsites. **b**, Native HvExoI perfused with 3dGlc. Glc (carbons: yellow sticks) and 3dGlc (carbons: orange sticks) at 1.0 occupancies are bound in -1 and +1 subsites. **c**, Native HvExoI perfused with octyl-O-Glc. Octyl-O-Glc (carbons: orange sticks) at 1.0 occupancy is bound across -1 and +1 subsites. **d**, Native HvExoI perfused with PEG (n=5-10). Two PEG molecules (PEG 1 in alternate conformations at occupancies 0.5, PEG 2 at occupancy 1.0) (carbons: orange sticks) are bound at the +1 and putative +2 subsites. Molecular surface morphologies are coloured by electrostatic potentials: white, neutral; blue, +5 $kT \cdot e^{-1}$; red, -5 $kT \cdot e^{-1}$. Grey, red, and blue represent carbon, oxygen and nitrogen atoms, respectively. Water molecules are shown as red or magenta (alternate water molecules) spheres in complexes with Glc and PEG molecules. Residues are marked in top left panel only.

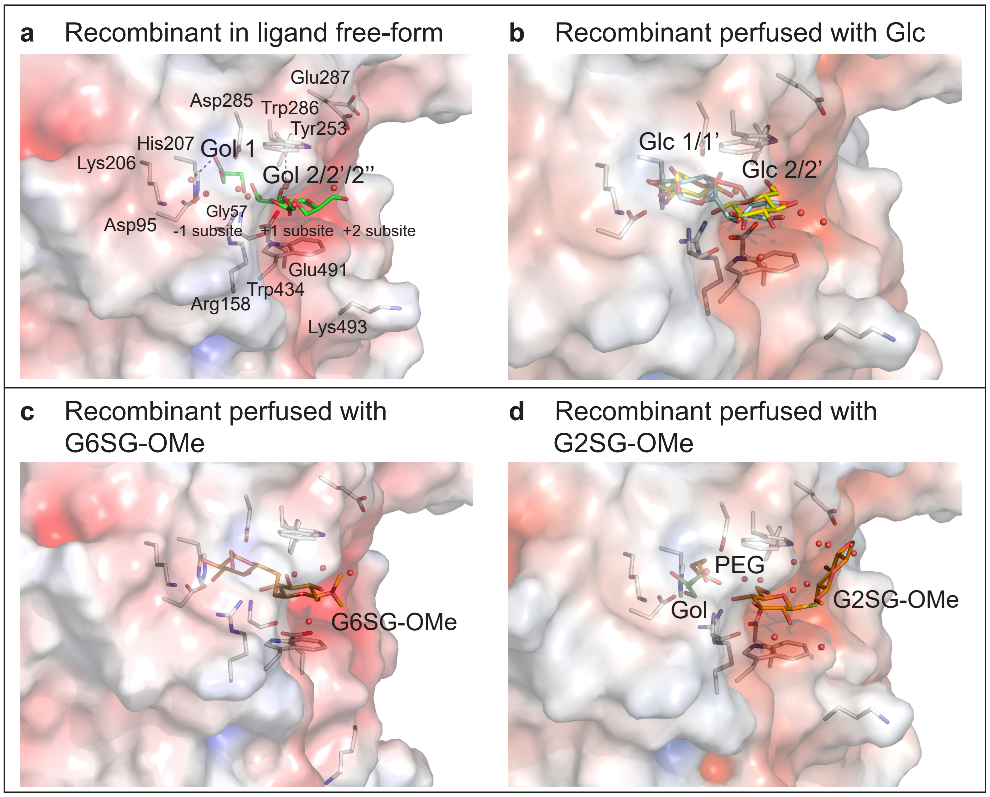

Supplementary Figure 2. Recombinant HvExoI in a ligand free-form, and with perfused Glc, G6SG-OMe or G2SG-OMe.

a, Ligand-free recombinant HvExoI. Two glycerol molecules (carbons: green sticks) (Gol 1 and Gol 2 in three alternate positions at occupancy 0.5) are in the -1 and +1 subsites. **b**, Recombinant HvExoI perfused with Glc. Two Glc molecules at 0.8 ($^4C_1$) (carbons: yellow sticks) and 0.2 ($^1S_3$) (carbons: cyan sticks) occupancies are shown in the -1 and +1 subsites, respectively. **c**, Recombinant HvExoI perfused with G6SG-OMe. G6SG-OMe (carbons: orange sticks) at 1.0 occupancy is bound across the -1 and +1 subsites. **d**, Recombinant HvExoI perfused with G2SG-OMe. G2SG-OMe (carbons: orange sticks) at 0.7 occupancy is bound across the +1 and putative +2 subsites. Glycerol (carbons: green sticks) and PEG (carbons: orange sticks) molecules fill-in the -1 subsite. Water molecules are shown as red spheres. Molecular surface morphologies are coloured by electrostatic potentials: white, neutral; blue, +5 $kT \cdot e^{-1}$; red, -5 $kT \cdot e^{-1}$. Grey, red, and blue represent carbon, oxygen and nitrogen atoms, respectively. Water molecules are shown as red spheres. Residues are marked in top left panel only.

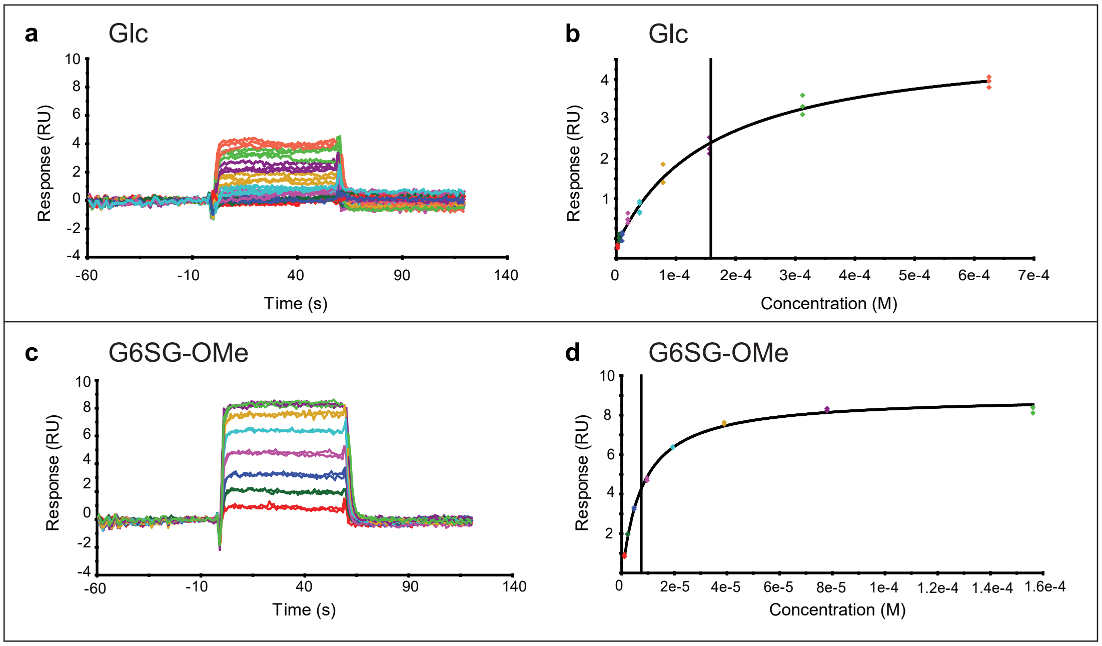

Supplementary Figure 3. SPR analyses of G6SG-OMe or Glc binding to recombinant HvExoI.

Sensograms of binding for **a**, Glc, and **c**, for G6SG-OMe. Steady state affinity binding curves for **b**, Glc, and **d**, for G6SG-OMe. Derived K_D values are 0.16 x 10^{-3} M for Glc and 0.008 x 10^{-3} M for G6SG-OMe. Triplicate experiments were performed in each case.

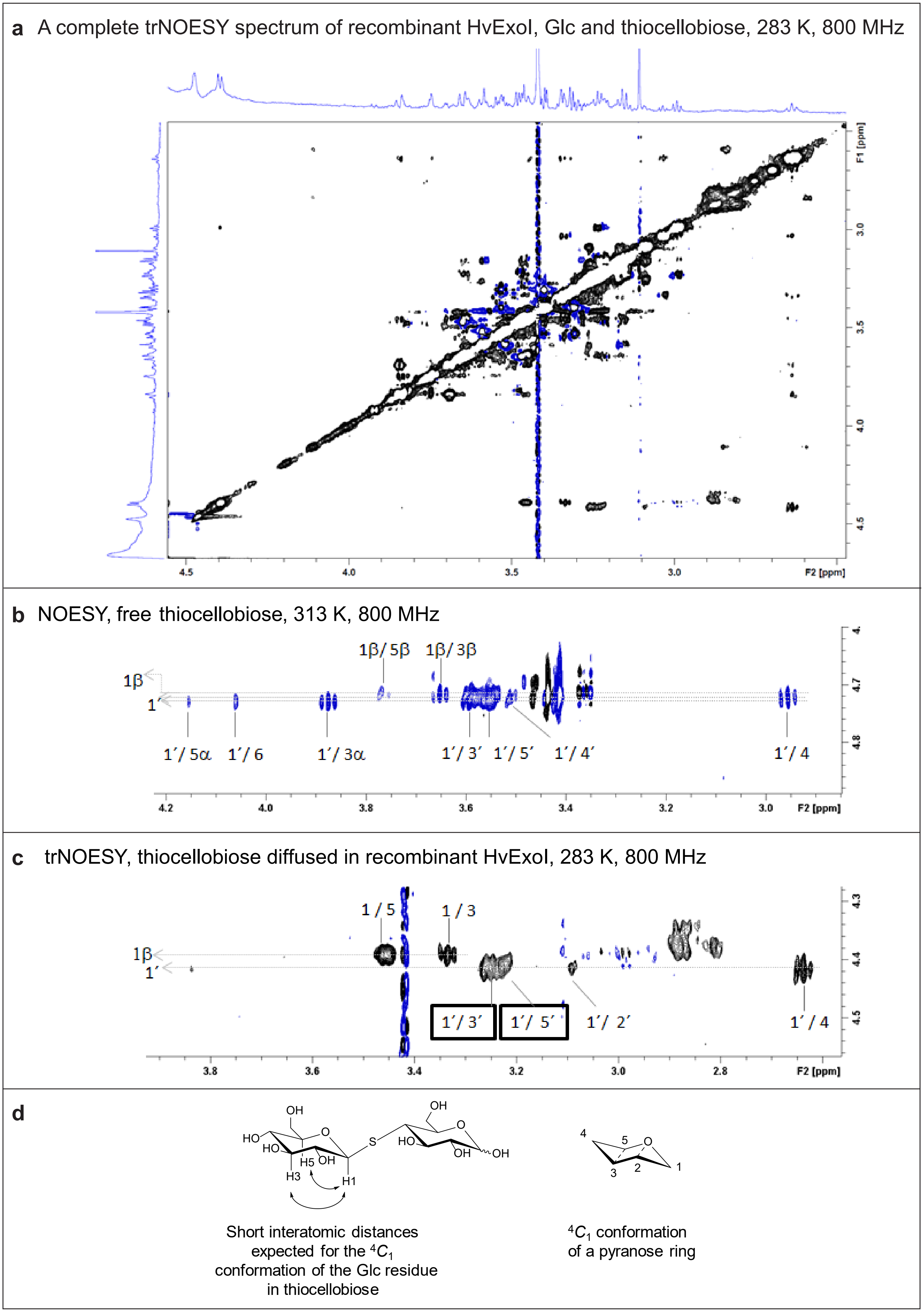
a A complete trNOESY spectrum of recombinant HvExoI, Glc and thiocellobiose, 283 K, 800 MHz
F1 [ppm]
F2 [ppm]
b NOESY, free thiocellobiose, 313 K, 800 MHz
1β/5β 1β/3β
1β
1′
1′/5α 1′/6 1′/3α 1′/3′ 1′/5′ 1′/4′ 1′/4
c trNOESY, thiocellobiose diffused in recombinant HvExoI, 283 K, 800 MHz
1/5 1/3
1′/3′ 1′/5′ 1′/2′ 1′/4
d
OH
HO
HO
H5 OH
H3
H1
O
S
HO
OH
OH
4
5
3
2
1
Short interatomic distances expected for the ⁴C₁ conformation of the Glc residue in thiocellobiose
⁴C₁ conformation of a pyranose ring

Supplementary Figure 4. Recombinant HvExoI binds thiocellobiose in the $^4C_1$ conformation at the -1 subsite without distortion of a pyranose ring.

a, Complete trNOESY spectrum of 0.057 mM recombinant HvExoI incubated with 0.285 mM Glc and 0.17 mM thiocellobiose was acquired with 300 msec mixing time at 283 K and 800 MHz. **b**, NOESY spectrum of free thiocellobiose with 600 msec mixing time was acquired at 313 K and 800 MHz. Cross-peaks correspond to positive NOEs. **c**, Enlargement of the trNOESY spectrum (panel a) in the same region as in panel b. Key negative trNOEs defining the $^4C_1$ conformation of bound thiocellobiose are annotated in squares. Blue lines in a and c refer to residual noise signals. **d**, Left: short interproton distances in the Glc residue of thiocellobiose (arrows), which adopts the $^4C_1$ conformation produce NOE and trNOE effects. Right: a pyranose ring with the co-planar C2-C3-C5-O atoms in the $^4C_1$ conformation.

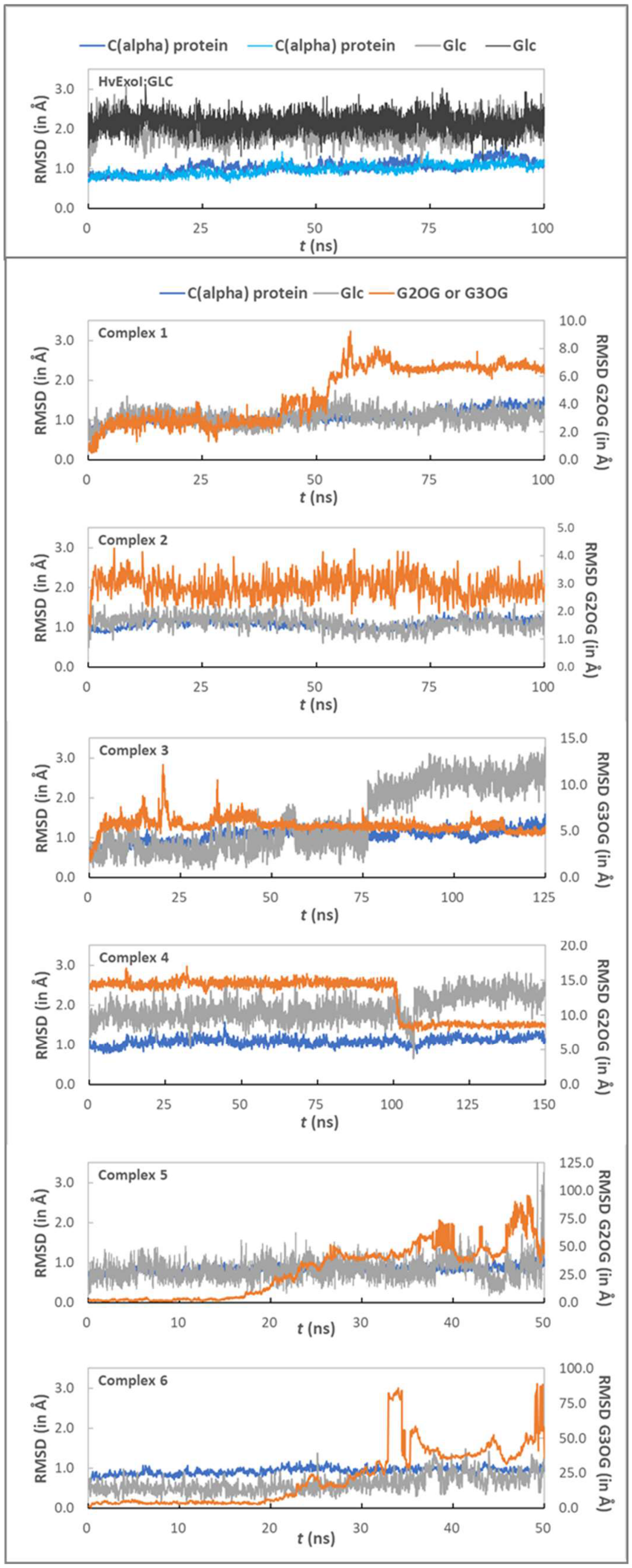

Supplementary Figure 5. Root-mean-square deviation (RMSD) values (Å) for Cα carbon atoms and ligands along MD simulations of HvExoI complexes.

RMSD values are specified for the HvExoI:Glc complex (top panel) or for HvExoI:Glc:G2OG (complexes 1-2 and 4-5) and HvExoI:Glc:G3OG (complexes 3 and 6) complexes, generated by docking.

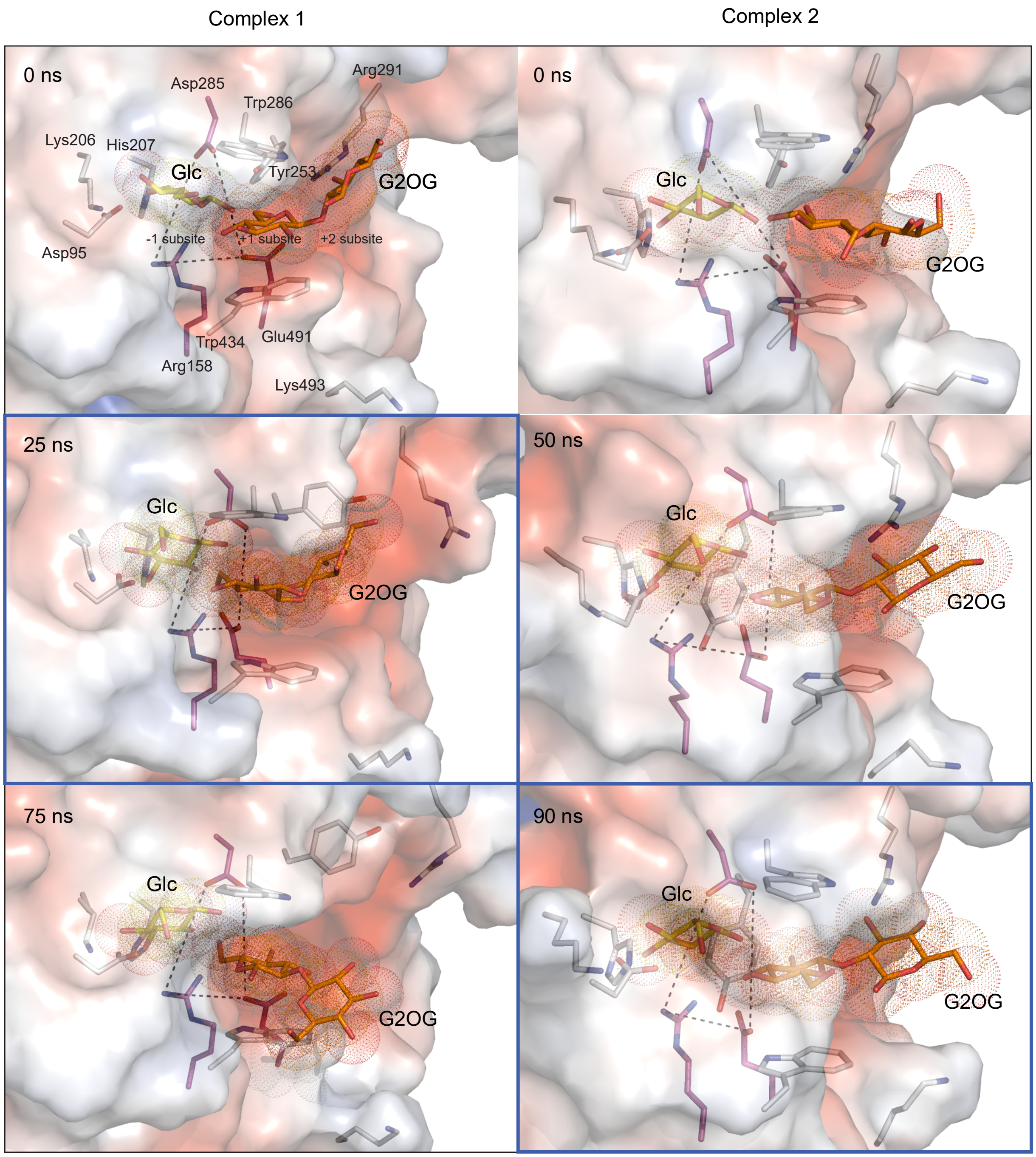

Supplementary Figure 6. Ternary HvExoI:Glc:G2OG complexes 1 (left) and 2 (right) along MD simulations after docking.

Selected residues (carbons: grey sticks), Glc in the -1 subsite (carbons: yellow sticks) and G2OG (carbons: orange sticks) are shown. Glc and G2OG molecules are presented with dots indicating van der Waals radii. Molecular surface morphologies are coloured by electrostatic potentials: white, neutral; blue, +5 kT·e^{-1}; red, -5 kT·e^{-1}. Frames highlighted in blue were used for PELE calculations to investigate the Glc displacement pathway. Structures in top to bottom panels were superposed with RMSD values between 0.769 and 0.859 Å (complex 1), and 0.813 and 1.287 Å (complex 2). Arg158-Glu491-Asp285 (carbons: purple sticks) toll-like barriers (dashed lines) are shown. Residues are marked in top left panel only.

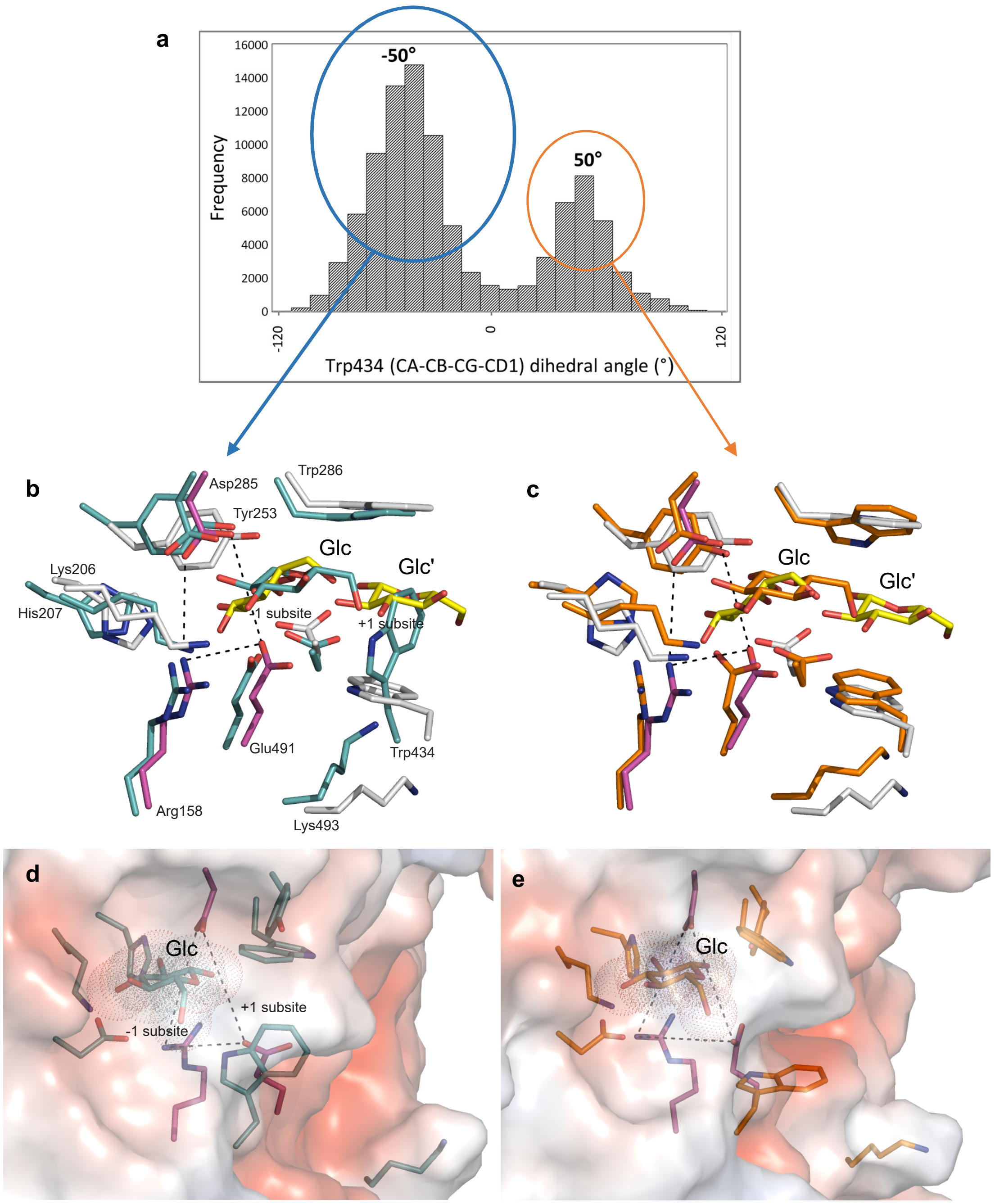

Supplementary Figure 7. MD simulations of the binary HvExoI:Glc complex.

a, Distribution of CA-CB-CG-CD1 dihedral angles of Trp434 show a major orientation at around -50°, and a second less-populated angle at around 50°. **b**, Active site residues are shown in the major orientation with Glc (carbons or residues and Glc: steel sticks), and **c**, in the minor orientation with Glc (carbons or residues and Glc: orange sticks), superposed on native HvExoI (PDB 3WLH; carbons of residues and Glc: grey and yellow sticks) with RMSD values of 0.96 Å (**b**), and 0.94 Å (**c**). **d-e**, same as **b**, **c** but with protein surface representations of binary HvExoI:Glc complexes coloured by electrostatic potentials: white, neutral; blue, +5 kT·e^{-1}; red, -5 kT·e^{-1}. Glc molecules are presented with dots indicating van der Waals radii. Left (**b-d**) and right (**c-e**) images are rotated by around 250° along y-axes. Arg158-Glu491-Asp285 (carbons: purple sticks) toll-like barriers (dashed lines) are shown in **c-e**. Residues are marked in **b** panel only.

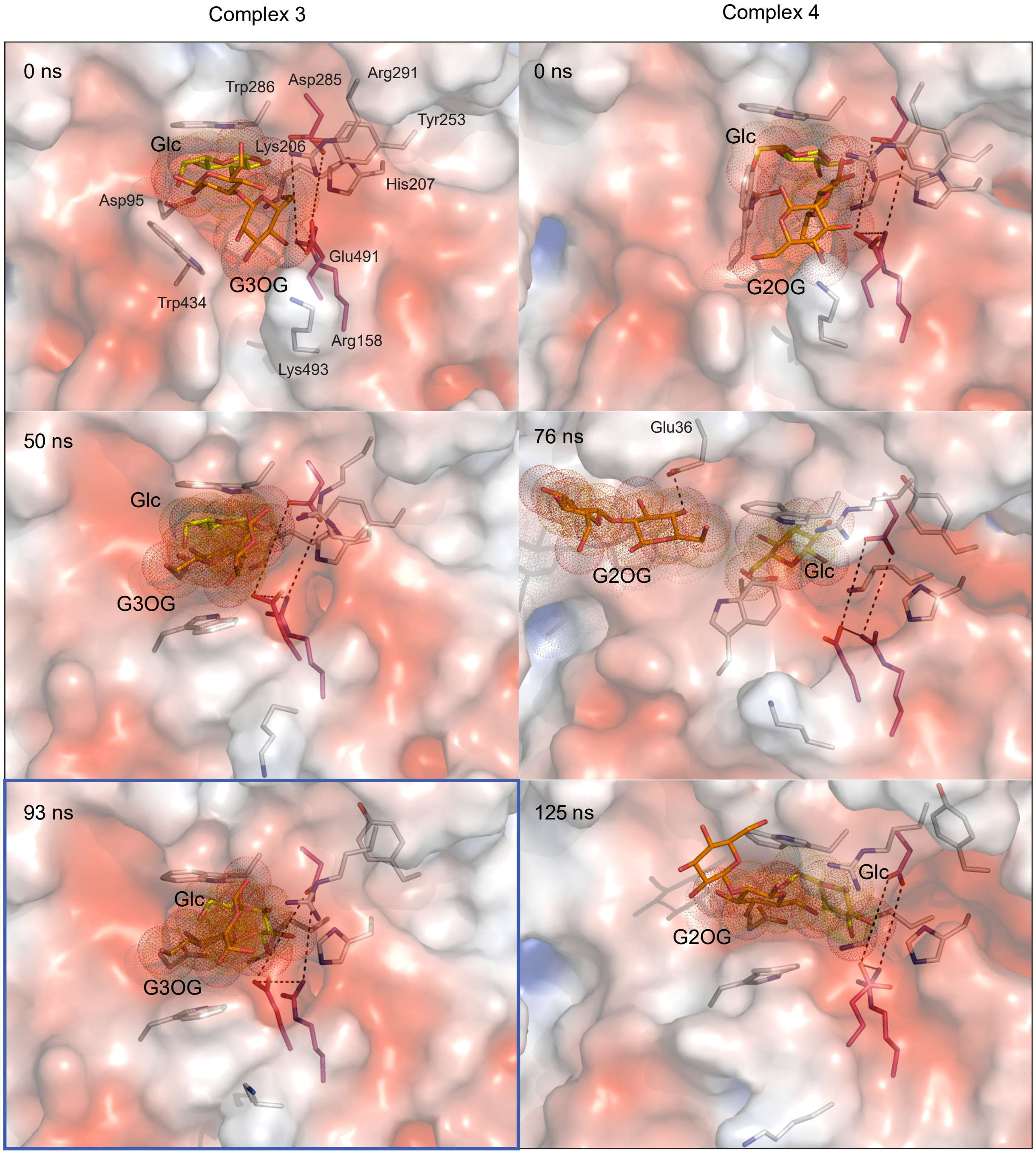

Supplementary Figure 8. Ternary HvExoI:Glc:G3OG complex 3 (left) and HvExoI:Glc:G2OG complex 4 (right) along MD simulations after docking.

Selected residues (carbons: grey sticks), Glc in the -1 subsite (carbons: yellow sticks) and G3OG or G2OG (carbons: orange sticks) are shown. Glc, G3OG and G2OG molecules are shown with dots indicating van der Waals radii. Molecular surface morphologies are coloured by electrostatic potentials: white, neutral; blue, +5 kT·e-1; red, -5 kT·e-1. The frame highlighted in blue was used for PELE calculations to investigate the Glc displacement pathway. Structures in top to bottom panels were superposed (Cα carbon atoms of 603 residues) with RMSD values between 0.982 and 1.021 Å (complex 3), and 0.819 and 0.973 Å (complex 4). Arg158-Glu491-Asp285 (carbons: purple sticks) toll-like barriers (dashed lines) are shown. Residues are marked in top left panel only.

a

b

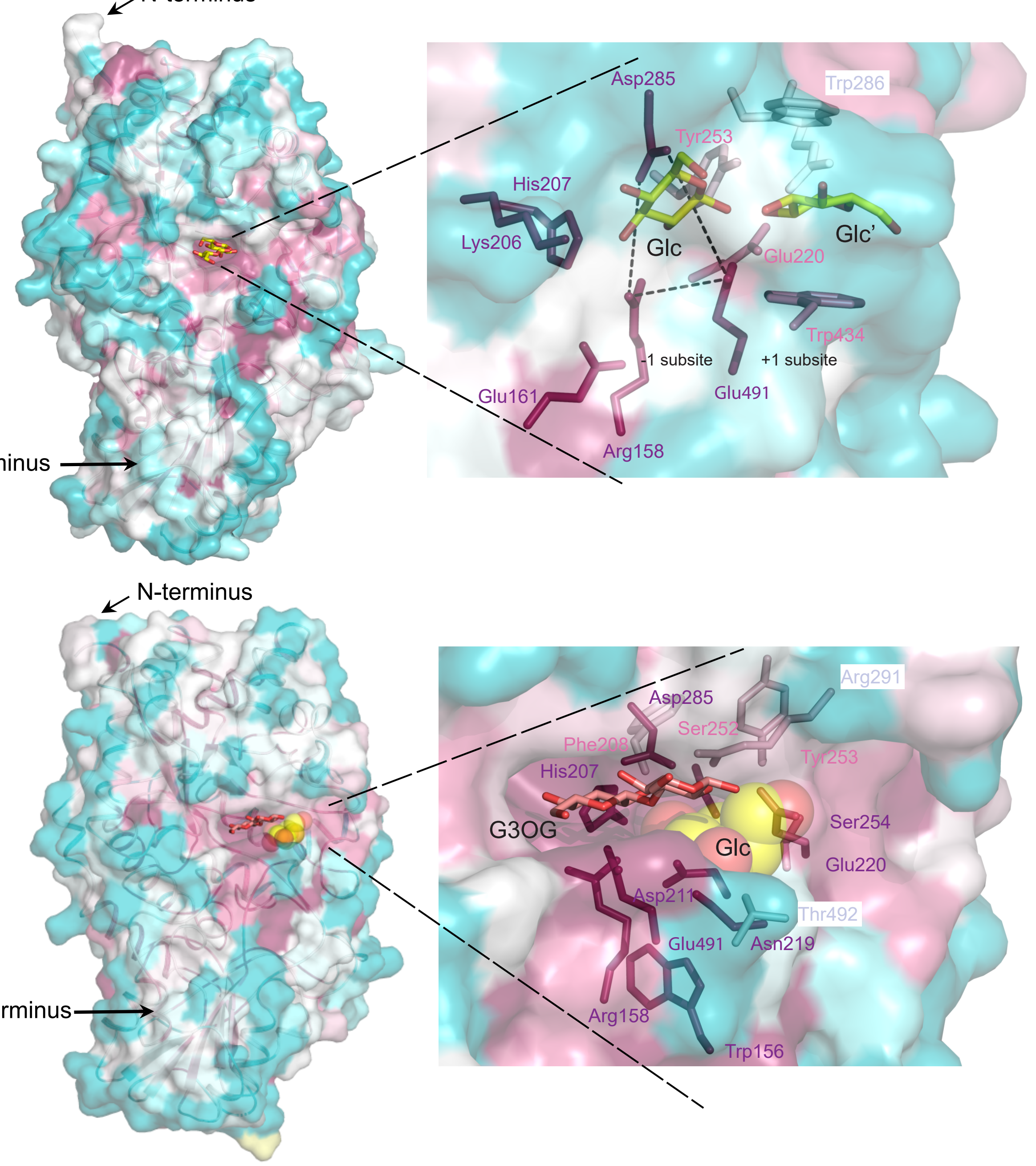

c

AAR	CIC	Variation in the active site:
Arg158	9,9	R, G
Glu161	9,9	E, R
Lys206	9,9	K
His207	9,9	H, Q
Glu220	9,8	E, R, T, Q, S, A, G, H
Tyr253	7,6	H, S, F, L, Y
Asp285	9,9	E, N, D
Trp286	6,5	Y, M, E, R, F, W, A, S
Arg291	6,5	H, G, S, Q, L, N, E, K, M, R, F, D, T
Trp434	9,8	W, G, M, K, R, H
Glu491	9,9	E, D

AAR	CIC	Variation in the lateral cavity:
Trp156	9,9	W, Y
Phe208	7,6	V, W, F, Y, L
Asp211	9,9	D, Y, E, G, T, L
Asn219	9,9	S, E, D, N
Ser252	9,9	G, A, N, S, T, C, D
Ser254	9,8	S, G, T, A, N, H,
Thr492	4,3	V, T, S, G, I, H, N, R, Y, W, M, F

Color scheme

1	2	3	4	5	6	7	8	9
Variable		Average				Conserved		

Insufficient data

AAR - Amino acid residue
CIC - Confidence interval colour
Residue conservation - 500 sequences with 35-95% sequence identity to HvExoI identified by ProMals3D were analysed.

Supplementary Figure 9. Conservation of amino acid residues (AAR) in HvExo1 classified in the GH3 family[3].

a, Conservation of surface residues of the HvExoI structure (left) (PDB 3WLH), and a detail of the active site with 11 residues (right: sticks coloured by confidence interval colour - CIC). **b**, Conservation of surface residues of the HvExoI:G3OG complex (left) (*cf.* **Fig. 6h**), and a detail of the lateral cavity with 14 residues (right; sticks coloured by CIC). **c**, Conservation scores[4] of residues in the active site (left), and in the lateral cavity (right). Scales of conservation represent the lower and upper bounds of intervals, where burgundy and turquois are the extremes of conservation on the scale 9=conserved and 1=variable, respectively. The HvExoI structure (PDB 3WLH) was used as a search parameter to identify 500 sequences with 35-95% sequence identity to the HvExo1 sequence, amongst them putative pro- and eukaryotic entries.

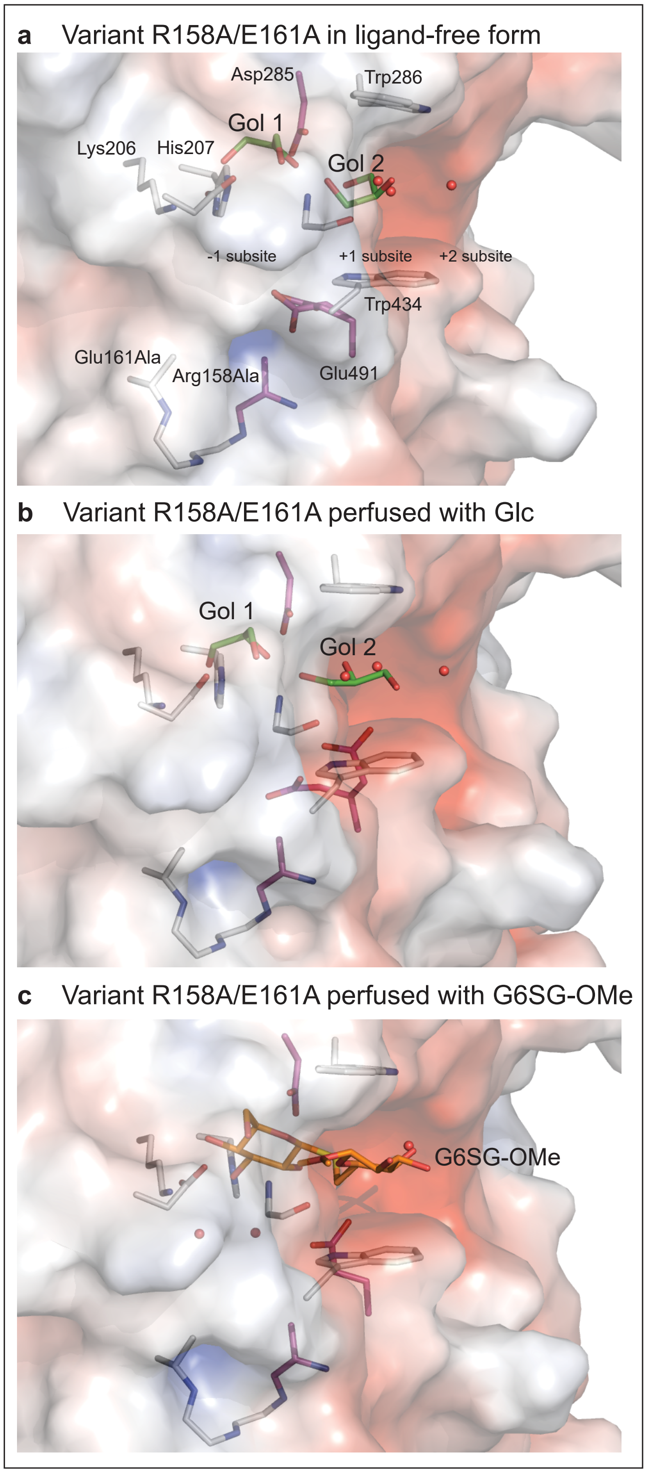

Supplementary Figure 10. Variant R158A/E161A HvExoI in a ligand-free form, and with perfused Glc or G6SG-OMe.

a, Ligand-free form of the R158A/E161A variant. Two glycerol molecules (carbons: green sticks) at 1.0 occupancies are bound in the -1 and +1 subsites. **b**, R158A/E161A perfused with Glc, which failed to bind; instead two glycerol molecules at 1.0 occupancies bound in the -1 and +1 subsites. **c**, R158A/E161A with bound G6SG-OMe (carbons: orange sticks) at 1.0 occupancy across the -1 and +1 subsites. Molecular surface morphologies are coloured by electrostatic potentials: white, neutral; blue, +5 kT·e-1; red, -5 kT·e-1. Grey, red, and blue represent carbon, oxygen and nitrogen atoms, respectively. Water molecules are shown as red spheres. Residues are marked in top panel only.

Supplementary Table 1. Chemical structures of reaction intermediates during organic synthesis, and chemical structures of ligands and inhibitors used in this work.

Compound	Chemical structure
Intermediate	
Methyl 6-thio-β-gentiobioside heptaacetate	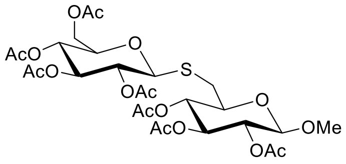
Methyl 3,4,6-tri-*O*-acetyl-2-*O*-trifluoromethanesulfonyl-β-D-mannopyranoside	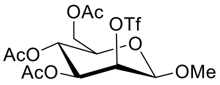
Methyl 2-thio-β-sophoroside heptaacetate	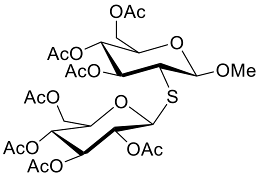
Ligand / inhibitor	
Glc (D-glucose)	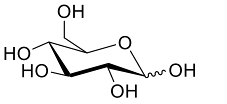
G6SG-OMe (methyl 6-thio-β-gentiobioside)	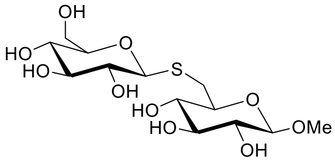
3dGlc (3-deoxy-D-glucose)	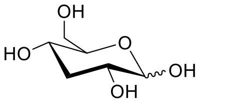
4dGlc (4-deoxy-D-glucose)	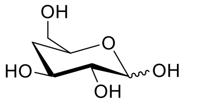
Octyl-O-Glc (n-octyl β-D-glucopyranoside)	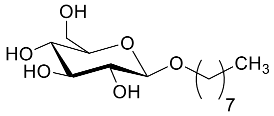
Octyl-S-Glc (n-octyl 1-thio-β-D-glucopyranoside)	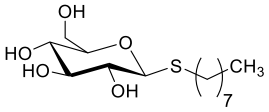
G2SG-OMe (methyl 2-thio-β-sophoroside)	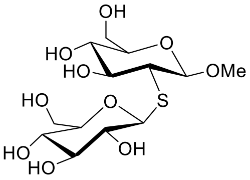

Supplementary Table 2. H-bonds of Glc with the protein or G2OG at representative points along the displacement pathway calculated by PELE for complex 1.

Separations from the Glc geometric centre in the -1 subsite are given. The analysis was performed with the FindHBond option of UCSFChimera, which uses a set of geometric criteria that consider different donor-acceptor combinations and are based on a survey of small-molecule crystal structures[5].

Donor		Acceptor		Hydrogen		D---A dist. (Å)	D-H---A dist. (Å)
Separations ~ 2-3 Å							
ARG158	NH1	GLC	O2	ARG158	HH12	2.777	1.792
ARG158	NH2	GLC	O3	ARG158	HH22	3.143	2.204
LYS206	NZ	GLC	O4	LYS206	HZ1	2.915	1.908
G2OG	O12	GLC	O1	G2OG	HO7	3.205	2.419
G2OG	O12	GLC	O5	G2OG	HO7	2.895	1.997
GLC	O1	ASP285	OD2	GLC	HO1	2.61	1.67
GLC	O2	ASP285	OD1	GLC	HO2	2.768	1.938
GLC	O3	HIS207	NE2	GLC	HO3	2.919	1.973
GLC	O4	ASP95	OD1	GLC	HO4	2.801	1.915
GLC	O6	ASP95	OD1	GLC	HO6	2.922	2.056
GLC	O6	ASP95	OD2	GLC	HO6	3.029	2.3
Separations ~ 5-7 Å							
TRP286	N	GLC	O4	TRP286	H	3.236	2.228
ARG291	NH1	GLC	O6	ARG291	HH12	3.228	2.333
G2OG	O4	GLC	O6	G2OG	HO4	2.741	1.81
GLC	O1	ASN219	OD1	GLC	HO1	3.303	2.819
GLC	O3	HIS207	NE2	GLC	HO3	2.828	1.862
Separations ~ 8-11 Å							
TRP156	NE1	GLC	O1	TRP156	HE1	2.946	2.101
GLU220	N	GLC	O5	GLU220	H	3.373	2.511
TYR253	N	GLC	O4	TYR253	H	2.922	1.946
GLC	O1	ASP211	O	GLC	HO1	3.198	2.235
GLC	O2	ASP211	OD1	GLC	HO2	2.683	1.737
GLC	O2	ASP211	OD2	GLC	HO2	2.944	2.206
GLC	O4	ASP285	OD2	GLC	HO4	2.735	1.994
GLC	O6	ASN219	OD1	GLC	HO6	2.703	1.745
Separations ~ 13-17 Å							
ASN219	ND2	GLC	O6	ASN219	HD21	2.965	2.037
GLC	O3	GLU220	OE2	GLC	HO3	3.192	2.493
GLC	O6	GLU491	O	GLC	HO6	2.764	1.849

Supplementary Table 3. H-bonds of Glc with the protein or G2OG at representative points along the displacement pathway calculated by PELE for complex 2.

Separations from the Glc geometric centre in the -1 subsite are given. The analysis was performed with the FindHBond option of UCSFChimera, which uses a set of geometric criteria that consider different donor-acceptor combinations and are based on a survey of small-molecule crystal structures[5].

Donor		Acceptor		**Hydrogen**		**D---A dist. (Å)**	**D-H---A dist. (Å)**
Separation ~ 2-3 Å							
G2OG	O10	GLC	O6	G2OG	HO5	2.867	1.933
G2OG	O8	GLC	O1	G2OG	HO8	2.88	1.92
G2OG	O9	GLC	O5	G2OG	HO9	2.814	1.854
GLC	O2	ASP285	OD2	GLC	HO2	2.89	1.931
GLC	O3	HIS207	NE2	GLC	HO3	2.98	2.13
GLC	O4	ASP95	OD1	GLC	HO4	2.518	1.58
GLC	O4	ASP95	OD2	GLC	HO4	3.273	2.566
GLC	O6	ASP95	OD2	GLC	HO6	2.564	1.624
ARG158	NH1	GLC	O2	ARG158	HH11	3.085	2.152
ARG158	NH2	GLC	O3	ARG158	HH22	3.221	2.355
LYS206	NZ	GLC	O3	LYS206	HZ2	2.741	1.756
Separation ~ 5-7 Å							
GLC	O1	ASP285	OD1	GLC	HO1	2.876	2.063
GLC	O1	TRP286	O	GLC	HO1	3.165	2.643
GLC	O2	ASP285	OD2	GLC	HO2	2.529	1.562
ARG158	NH1	GLC	O3	ARG158	HH11	3.251	2.413
ARG158	NH2	GLC	O3	ARG158	HH22	3.330	2.503
TRP286	N	GLC	O2	TRP286	H	3.192	2.259
ARG291	NH2	GLC	O1	ARG291	HH22	2.879	1.863
GLU491	OE2	GLC	O4	GLU491	HE2	2.568	1.612
THR492	OG1	GLC	O6	THR492	HG1	2.835	1.923
Separation ~ 8-11 Å							
G2OG	O8	GLC	O1	G2OG	HO8	2.892	1.916
G2OG	O8	GLC	O5	G2OG	HO8	2.881	2.261
GLC	O1	G2OG	O9	GLC	HO1	2.679	1.72
GLC	O2	ASP285	OD2	GLC	HO2	2.725	1.774
GLC	O6	G2OG	O8	GLC	HO6	3.335	2.374
THR492	OG1	GLC	O4	THR492	HG1	2.708	1.796
Separation ~ 13-17 Å							
GLC	O1	G2OG	O4	GLC	HO1	2.765	1.819
GLC	O4	GLC	O6	GLC	HO4	2.803	1.938
LYS493	NZ	GLC	O2	LYS493	HZ2	3.007	2.142

Supplementary Table 4. H-bonds of Glc with the protein or G3OG at representative points along the displacement pathway calculated by PELE for complex 3.

Separations from the Glc geometric centre in the -1 subsite are given. The analysis was performed with the FindHBond option of UCSFChimera, which uses a set of geometric criteria that consider different donor-acceptor combinations and are based on a survey of small-molecule crystal structures[5].

Donor		Acceptor		**Hydrogen**		**D---A dist. (Å)**	**D-H---A dist. (Å)**
Separations ~ 2-3 Å							
ARG158	NH1	GLC	O2	ARG158	HH12	3.002	2.033
ARG158	NH2	GLC	O3	ARG158	HH22	3.008	2.057
LYS206	NZ	GLC	O3	LYS206	HZ1	3.121	2.253
LYS206	NZ	GLC	O4	LYS206	HZ1	3.224	2.408
TRP286	N	GLC	O1	TRP286	H	3.303	2.326
GLC	O1	ASP285	OD1	GLC	HO1	3.137	2.468
GLC	O1	ASP285	OD2	GLC	HO1	2.689	1.899
GLC	O2	ASP285	OD1	GLC	HO2	2.632	1.675
GLC	O4	ASP95	OD1	GLC	HO4	2.566	1.629
GLC	O6	ASP95	OD2	GLC	HO6	2.618	1.684
Separations ~ 5-7 Å							
TRP286	N	GLC	O6	TRP286	H	2.804	1.8
G3OG	O2	GLC	O1	G3OG	H4	2.964	2.114
G3OG	O2	GLC	O5	G3OG	H4	3.351	2.567
G3OG	O3	GLC	O5	G3OG	H6	2.729	1.853
GLC	O3	ASN219	OD1	GLC	HO3	2.704	1.805
Separations ~ 8-11 Å							
SER252	OG	GLC	O2	SER252	HG	2.76	1.829
TYR253	N	GLC	O1	TYR253	H	3.06	2.173
ARG291	NH1	GLC	O6	ARG291	HH11	2.903	2.103
GLC	O2	ASP285	OD1	BGC	HO2	3.326	2.516
GLC	O2	ASP285	OD2	BGC	HO2	2.76	1.84
Separations ~ 13-17 Å							
G3OG	O2	GLC	O2	G3OG	H4	2.768	1.827
GLC	O1	THR492	OG1	GLC	HO1	2.777	1.824
GLC	O3	GLU491	OE1	GLC	HO3	2.895	2.04
GLC	O6	GLU220	OE1	GLC	HO6	2.952	2.054
Separations ~ 13-17 Å							
ALA296	N	GLC	O6	ALA296	H	2.883	1.907
G3OG	O6	GLC	O3	G3OG	H13	2.796	1.867
GLC	O1	GLU220	OE1	GLC	HO1	2.852	1.988
GLC	O2	GLU220	OE1	GLC	HO2	2.815	1.921
GLC	O3	G3OG2	O8	GLC	HO3	2.751	1.901

Supplementary Table 5. Parameters of 22 crystal structures of native HvExoI with entrapped Glc and in complex with ligands, and recombinant WT and variant HvExoI in apo-forms and in complex with ligands.

PDB accession	HvExoI	Ligand soaked [a]	Ligand bound at subsite [b] -1 +1	a=b [d] Å	c [d] Å	Reference
Native HvExoI						
3WLH	WT	none	Glc ↔ Glc [c]	101.02	182.06	This work
3WLJ	WT	3dGlc	Glc 3dGlc	100.82	181.65	This work
3WLK	WT	4dGlc	Glc empty	100.56	181.76	This work
3WLL	WT	PEG400	empty PEG	100.27	181.70	This work
3WLM	WT	octyl-O-Glc	Octyl-O-Glc	101.02	180.80	This work
3WLN	WT	octyl-S-Glc	Octyl-S-Glc	100.70	181.88	This work
Recombinant HvExoI						
3WLI	WT	none	Gol Gol	99.22	183.46	This work
3WLO	WT	Glc	Glc Glc	100.16	183.01	This work
3WLP	WT	G6SG-OMe	G6SG-OMe	99.94	182.87	This work
6MD6	WT	G2SG-OMe	G2SG-OMe	100.31	182.15	This work
3WLQ	R158A/E161A	none	Gol Gol	100.78	183.29	This work
3WLR	R158A/E161A	Glc	Gol Gol	100.69	183.45	This work
6MI1	R158A/E161A	G6SG-OMe	G6SG-OMe	101.72	182.08	This work
Other HvExoI structures deposited in PDB						
1EX1	WT	none	Glc	102.09	184.50	6
1IEQ	WT	Glc	Glc	100.95	181.20	7
1IEV	WT	conduritol B epoxide	hexitol	101.01	181.55	7
1IEW	WT	DNP-2d-2F-Glc	2d-2F-Glc	100.96	181.25	7
1IEX	WT	G4SG4OG4SG4OG4SG	G4SGO	102.42	185.48	7
1J8V	WT	G3SGOG-4NP	G3SGO	100.53	181.96	1
1LQ2	WT	PheGlcIm	PheGlcIm	100.69	182.68	8
1X38	WT	PheGlcIm	PheGlcIm	100.55	182.41	9
1X39	WT	AmGlcIm	AmGlcIm	99.99	184.39	9

[a] 3-Deoxy-glucose (3dGlc), 4-deoxy-glucose (4dGlc), polyethylene glycol 400 (PEG400), n-octyl-O-β-D-glucopyranoside (octyl-O-Glc), n-octyl-1-thio-β-D-glucopyranoside (octyl-S-Glc), glucose (Glc), methyl 6-thio-β-gentiobioside (G6SG-OMe), methyl 2-thio-β-sophoroside (G2SG-OMe), conduritol B epoxide, 2,4-dinitrophenyl 2-deoxy-2-fluoro β-D-glucopyranoside (DNP-2d-2F-Glc), 4^{I}, 4^{III}, 4^{V}-S-trithiocellohexaose (G4SG4OG4SG4OG4SG), 4-nitrophenyl *S*-(β-D-glucopyranosyl)-(1,3)-(3-thio-β-D-glucopyranosyl)-(1,3)-β-D-glucopyranoside (G3SG3OG-4NP), glucophenyl imidazole (PheGlcIm), anilinomethyl glucoimidazole (AmGlcIm).

[b] When identified, PEG400 and glycerol molecules were not assigned to -1 and +1 subsites, except 3WLL, 3WLI, 3WLQ and 3WLR.

[c] Glc oscillates between -1 and +1 subsites.

[d] Tetragonal $P4_32_12$ unit cell angles α, β, and γ were 90 degrees.

Supplementary Table 6. List of primers used to construct the R158A/E161A variant of HvExoI for heterologous expression in *P. pastoris*.

Variant [a]	Sequences (5' to 3') and notes [b]
R158A/E161A	F:GAGATCCAAGATGGGGT*GCT*TGTTACG*CT*TCTTACTCTGAAGATAGAAGAATTGT<br>R:ACAATTCTTCTATCTTCAGAGTAAGA*AG*CGTAACA*AGC*ACCCCATCTTGGATCTC

[a] DNA fusion was sequenced in both directions and was identified to be correct.

[b] F and R indicate forward and reverse primers, respectively. Mutations are highlighted in red italics.

Supplementary Table 7. Data collection and refinement statistics of crystal structures of native HvExoI with entrapped Glc and in complex with 3dGlc and 4dGlc.

	With trapped Glc	3dGlc	4dGlc
Data collection			
Space group	$P4_32_12$	$P4_32_12$	$P4_32_12$
Cell dimensions			
a, *b*, *c* (Å)	101.02, 101.02, 182.06	100.82, 100.82, 181.65	100.56, 100.56, 181.76
α, β, γ (°)	90.00, 90.00, 90.00	90.00, 90.00, 90.00	90.00, 90.00, 90.00
Resolution (Å)*	31.94-1.65 (1.69-1.65)	34.18-1.67 (1.72-1.67)	88.05-1.80 (1.86-1.80)
R_{merge}	8.9	5.2	9.3
$I / \sigma I$ *	34.1 (1.0)	57.8 (2.7)	28.0 (1.3)
Completeness (%)*	98.5 (92.5)	99.0 (94.7)	99.5 (93.4)
Redundancy	13	13	14
Refinement			
Resolution (Å)*	31.94-1.65 (1.69-1.65)	34.18-1.67 (1.72-1.67)	88.05-1.80 (1.86-1.80)
No. reflections	106290	101919	82,188
R_{work} / R_{free}	16.1/19.3	15.9/18.5	15.0/17.4
No. atoms			
Protein	4,606	4,610	4,789
Ligand/ion	24	23	12
Water	826	830	875
B-factors			
Protein	22.7	22.3	38.8
Ligand/ion	32.4	34.4	31.3
Water	40.1	41.3	44.3
R.m.s. deviations			
Bond lengths (Å)	0.018	0.013	0.013
Bond angles (°)	1.587	1.408	1.327

* Values in parentheses are for highest-resolution shell.

Supplementary Table 8. Data collection and refinement statistics of crystal structures of native HvExoI in complex with PEG400, octyl-O-Glc and octyl-S-glucoside.

	PEG400	Octyl-O-Glc	Octyl-S-Glc
Data collection			
Space group	$P4_32_12$	$P4_32_12$	$P4_32_12$
Cell dimensions			
a, *b*, *c* (Å)	100.27, 100.27, 181.70	101.02, 101.02, 180.80	100.70, 100.70, 181.88
α, β, γ (°)	90.00, 90.00, 90.00	90.00, 90.00, 90.00	90.00, 90.00, 90.00
Resolution (Å)*	87.71-1.80 (1.85-1.80)	42.26-1.90 (1.95-1.90)	88.05-2.00 (2.05-2.00)
R_{merge}	10.1	20.4	16.7
$I / \sigma I$ *	14.9 (1.1)	10.1 (1.0)	12.0 (1.3)
Completeness (%)*	99.2 (97.4)	100 (99.5)	99.9 (98.7)
Redundancy	6	12	14
Refinement			
Resolution (Å)*	87.71-1.80 (1.85-1.80)	42.26-1.90 (1.95-1.90)	88.05-2.00 (2.05-2.00)
No. reflections	81,336	70,533	60,745
R_{work} / R_{free}	16.1/19.1	17.0/20.1	16.9/20.5
No. atoms			
Protein	4,566	4,586	4,590
Ligand/ion	-	20	20
Water	883	870	901
B-factors			
Protein	33.6	33.2	40.3
Ligand/ion	-	34.5	48.2
Water	46.2	42.6	43.6
R.m.s. deviations			
Bond lengths (Å)	0.011	0.010	0.011
Bond angles (°)	1.253	1.266	1.372

* Values in parentheses are for highest-resolution shell.

Supplementary Table 9. Data collection and refinement statistics of crystal structures of recombinant WT HvExoI in a ligand-free form and in complex with Glc, G6SG-OMe and G2SG-OMe.

	Ligand-free	Glc	G6SG-OMe	G2SG-OMe
Data collection				
Space group	$P4_32_12$	$P4_32_12$	$P4_32_12$	$P4_32_12$
Cell dimensions				
a, *b*, *c* (Å)	99.22, 99.22, 183.46	100.16, 100.16, 183.01	99.94, 99.94, 182.87	100.31, 100.31, 182.15
α, β, γ (°)	90.00, 90.00, 90.00	90.00, 90.00, 90.00	90.00, 90.00, 90.00	90.00, 90.00, 90.00
Resolution (Å)*	87.27-1.45 (1.49-1.45)	87.86-1.55 (1.59-1.55)	25.41-1.57 (1.61-1.57)	46.13-1.68 (1.72-1.68)
R_{merge}	6.3	12.4	5.5	7.6
$I / \sigma I$ *	60.0 (2.3)	49.0 (1.8)	68.4 (6.6)	39.2 (4.0)
Completeness (%)*	99.3 (99.5)	99.5 (97.6)	97.0 (99.2)	99.9 (99.3)
Redundancy	26	27	28	29
Refinement				
Resolution (Å)*	87.27-1.45 (1.49-1.45)	87.86-1.55 (1.59-1.55)	25.41-1.57 (1.61-1.57)	46.13-1.68 (1.72-1.68)
No. reflections	152,633	127,330	118,576	101,119
R_{work} / R_{free}	14.6/16.3	16.7/19.0	16.2/18.1	13.2/15.9
No. atoms				
Protein	4,664	4,622	4,626	5,825
Ligand/ion	-	24	24	24
Water	894	1025	853	1003
B-factors				
Protein	18.1	20.1	17.6	19.4
Ligand/ion	-	19.4	15.9	36.2
Water	36.4	37.5	36.6	38.4
R.m.s. deviations				
Bond lengths (Å)	0.019	0.021	0.016	0.020
Bond angles (°)	1.753	1.845	1.761	2.282

* Values in parentheses are for highest-resolution shell.

Supplementary Table 10. Data collection and refinement statistics of crystal structures of the R158A/E161A HvExoI variant in the ligand-free form and in complex with Glc and G6SG-OMe.

	Ligand-free	Glc	G6SG-OMe
Data collection			
Space group	$P4_32_12$	$P4_32_12$	$P4_32_12$
Cell dimensions			
a, *b*, *c* (Å)	100.78, 100.78, 183.16	100.69, 100.69, 183.29	101.72, 101.72, 183.45
α, β, γ (°)	90.00, 90.00, 90.00	90.00, 90.00, 90.00	90.00, 90.00, 90.00
Resolution (Å)*	88.30-1.65 (1.69-1.65)	46.37-2.21 (2.26-2.21)	88.96-2.30 (2.36-2.30)
R_{merge}	5.6	16.7	11.9
$I / \sigma I$ *	58.7 (2.3)	29.1 (2.4)	30.1 (2.0)
Completeness (%)*	99.8 (98.3)	98.8 (88.1)	99.3 (99.5)
Redundancy	26	25	29
Refinement			
Resolution (Å)*	88.30-1.65 (1.69-1.65)	46.37-2.21 (2.26-2.21)	88.96-2.30 (2.36-2.30)
No. reflections	107,601	45,206	41,138
R_{work} / R_{free}	15.7/18.8	16.9/17.2	16.8/22.4
No. atoms			
Protein	4,626	4,621	4,598
Ligand/ion	-	-	24
Water	1005	562	330
B-factors			
Protein	24.7	49.3	36.2
Ligand/ion	-	-	50.3
Water	43.0	58.1	45.3
R.m.s. deviations			
Bond lengths (Å)	0.020	0.020	0.019
Bond angles (°)	1.715	1.716	2.081

* Values in parentheses are for highest-resolution shell.

Supplementary References

1. Hrmova, M., DeGori, R., Smith, B. J., Fairweather, J. K., Driguez, H. et al. Structural basis for a broad specificity in higher plant β-D-glucan glucohydrolases. *Plant Cell* **14**, 1-22 (2002).
2. Fernandez-Alonso, M. C., Canada, F. J., Jimenez-Barbero, J. & Cuevas, G. Molecular recognition of saccharides by proteins. Insights on the origin of the carbohydrate-aromatic interactions. *J. Am. Chem. Soc.* **127**, 7379-7386 (2005).
3. Lombard, V., Golaconda Ramulu, H., Drula, E., Coutinho, P. M. & Henrissat, B. The carbohydrate-active enzymes database (CAZy) in 2013. *Nucleic Acids Res.* **42D**, 490-495 (2014).
4. Celniker, G., Nimrod, G., Ashkenazy, H., Glaser, F., Martz, E. et al. ConSurf: using evolutionary data to raise testable hypotheses about protein function. *Isr. J. Chem.* **53**, 199-206 (2013).

   Mills, J. E. & Dean, P. M. Three-dimensional hydrogen-bond geometry and probability information from a crystal survey. *J. Comput. Aided Mol. Des.* **10**, 607-622 (1966).
5. Mills, J. E. & Dean, P. M. Three-dimensional hydrogen-bond geometry and probability information from a crystal survey. *J. Comput. Aided Mol. Des.* **10**, 607-622 (1966).
6. Varghese, J. N., Hrmova, M. & Fincher, G. B. Three-dimensional structure of a barley β-D-glucan exohydrolase, a family 3 glycosyl hydrolase. *Structure* **7**, 179-190 (1999).
7. Hrmova, M. Varghese, J. N., DeGori, R., Smith, B. J., Driguez, H. *et al.* Catalytic mechanisms and reaction intermediates along the hydrolytic pathway of plant β-D-glucan glucohydrolase. *Structure* **9**, 1005-1016 (2001).
8. Hrmova, M., De Gori, R., Smith, B. J., Vasella, A., Varghese, J. N., et al. Three-dimensional structure of the barley β-D-glucan glucohydrolase in complex with a transition-state mimic. *J. Biol. Chem.* **279**, 4970-4980 (2004).
9. Hrmova, M., Streltsov, V. A., Smith, B. J., Vasella, A., Varghese, J. N. et al. Structural rationale for low nanomolar binding of transition state mimics to a family GH3 β-D-glucan glucohydrolase from barley. *Biochemistry (USA)* **44**, 16529-16539 (2005).
