# Supplementary material for: Discovery of processive catalysis by an exo-hydrolase with a pocket-shaped active site
Source: Nat Commun. 2019 May 20;10:2222. doi: 10.1038/s41467-019-09691-z (PMC6527550; doi:10.1038/s41467-019-09691-z)
Supplement: Supplementary file 3 — Description of Additional Supplementary Files [file 41467_2019_9691_MOESM3_ESM.pdf]

Description of Supplementary Files

File Name: Supplementary Data 1.

Description: RMSD (root-mean-square deviation) Tables

RMSD HvExoI-Glc
RMSD Complex 1
RMSD Complex 2
RMSD Complex 3
RMSD Complex 4
RMSD Complex 5
RMSD Complex 6

File Name: Supplementary Data 2.

Description: MD (Molecular dynamics) of HvExoI-Glc, W434 dihedral (angle).

File Name: Supplementary Data 3.

Description: GaudiMM (Genetic Algorithms with Unrestricted Descriptors for Intuitive Molecular Modeling), X-ray_without_NMA (Normal Mode Algorithm)

GaudiMM, X-ray_with_NMA
GaudiMM, MD (Molecular Dynamics) without_NMA
GaudiMM, MD with_NMA

File Name: Supplementary Data 4.

Description: PELE (Protein Energy Landscape Exploration) data for complexes 1, 2, 3

Fig. 6d

File Name: Supplementary Movie 1.

Description: Molecular animation of the sequence of events involving the glucose product entrapment, incoming substrate binding and glucose displacement in a plant exo-hydrolase HvExoI, and how this sequence of events underlies substrate-product assisted processive catalysis. The movie shows the entrapped glucose molecule in the -1 subsite of the active site, through twelve residues located on seven loops. After the incoming β-D-glucopyranosyl-(1,3)-D-glucose substrate binds in the +1 and putative +2 subsites, the glucose product adjusts its binding patterns and traverses from the -1 subsite through rotations of Arg158 and Asp285 sidechains and associated backbone atoms, into the autonomous and transient lateral cavity, from where it advances through the aperture into the bulk solvent. The video was prepared in Chimera (Pettersen, E. F. et al. J. Comput. Chem. 25, 1605-1612; 2004), using the HD Movie Maker tool.
