# Supplementary material for: Discovery of processive catalysis by an exo-hydrolase with a pocket-shaped active site
Source: Nat Commun. 2019 May 20;10:2222. doi: 10.1038/s41467-019-09691-z (PMC6527550; doi:10.1038/s41467-019-09691-z)
Supplement: Supplementary file 9 — Reporting Summary [file 41467_2019_9691_MOESM9_ESM.pdf]

nature research

Corresponding author(s): Maria Hrmova

Last updated by author(s): Mar 21, 2019

Reporting Summary

Nature Research wishes to improve the reproducibility of the work that we publish. This form provides structure for consistency and transparency in reporting. For further information on Nature Research policies, see Authors & Referees and the Editorial Policy Checklist.

Statistics

For all statistical analyses, confirm that the following items are present in the figure legend, table legend, main text, or Methods section.

n/a	Confirmed	
☐	☒	The exact sample size (*n*) for each experimental group/condition, given as a discrete number and unit of measurement
☒	☐	A statement on whether measurements were taken from distinct samples or whether the same sample was measured repeatedly
☒	☐	The statistical test(s) used AND whether they are one- or two-sided<br>*Only common tests should be described solely by name; describe more complex techniques in the Methods section.*
☒	☐	A description of all covariates tested
☒	☐	A description of any assumptions or corrections, such as tests of normality and adjustment for multiple comparisons
☒	☐	A full description of the statistical parameters including central tendency (e.g. means) or other basic estimates (e.g. regression coefficient) AND variation (e.g. standard deviation) or associated estimates of uncertainty (e.g. confidence intervals)
☒	☐	For null hypothesis testing, the test statistic (e.g. *F*, *t*, *r*) with confidence intervals, effect sizes, degrees of freedom and *P* value noted<br>*Give P values as exact values whenever suitable.*
☒	☐	For Bayesian analysis, information on the choice of priors and Markov chain Monte Carlo settings
☒	☐	For hierarchical and complex designs, identification of the appropriate level for tests and full reporting of outcomes
☒	☐	Estimates of effect sizes (e.g. Cohen's *d*, Pearson's *r*), indicating how they were calculated

Our web collection on statistics for biologists contains articles on many of the points above.

Software and code

Policy information about availability of computer code

Data collection	All software applications used in the data collection are described and relevant references are cited.
Data analysis	Software applications described in Methods with associated references were used without code modifications. For analyses of geometrical parameters along MD simulations, we have used an in-house script, which is available upon request from the corresponding author.

For manuscripts utilizing custom algorithms or software that are central to the research but not yet described in published literature, software must be made available to editors/reviewers. We strongly encourage code deposition in a community repository (e.g. GitHub). See the Nature Research guidelines for submitting code & software for further information.

Data

Policy information about availability of data

All manuscripts must include a data availability statement. This statement should provide the following information, where applicable:

- Accession codes, unique identifiers, or web links for publicly available datasets
- A list of figures that have associated raw data
- A description of any restrictions on data availability

The 13 Protein Data Bank (PDB) accession codes of the solved crystal structures (3WLH, 3WLJ, 3WLK, 3WLM, 3WLN, 3WLL, 3WLI, 3WLO, 3WLP, 3WLQ, 3WLR, 6MD6, 6MI1) have been registered in the PDB, as stated under 'Data availability' in the Methods sections. The coordinate and structure files of 11 of these structures (3WLH, 3WLJ, 3WLK, 3WLM, 3WLN, 3WLL, 3WLI, 3WLO, 3WLP, 3WLQ, 3WLR) have been released and are freely available in the PDB. The coordinate and structure files of the two unreleased entries (6MD6, 6MI1) will be released upon the acceptance of the manuscript. The corresponding author will arrange the release of the two entries 6MD6 and 6MI1 in the PDB, after the press-release of the manuscript in Nature Communications.

Field-specific reporting

Please select the one below that is the best fit for your research. If you are not sure, read the appropriate sections before making your selection.

☒ Life sciences ☐ Behavioural & social sciences ☐ Ecological, evolutionary & environmental sciences

For a reference copy of the document with all sections, see nature.com/documents/nr-reporting-summary-flat.pdf

Life sciences study design

All studies must disclose on these points even when the disclosure is negative.

Sample size	For in vitro assays, sample sizes were determined empirically using our extended of experience using similar assays.
Data exclusions	No data were excluded from any other experiments.
Replication	In enzyme inhibition kinetic data, each inhibitor was tested at six concentrations at 0.4-3 times the Ki values in duplicate. In Surface Plasmon Resonance evaluations, for each interaction a serial dilution of an analyte covered the 10 x KD - 0.1 x KD concentrations.
Randomization	Covariates were not used in this study.
Blinding	Investigators were not blinded.

Reporting for specific materials, systems and methods

We require information from authors about some types of materials, experimental systems and methods used in many studies. Here, indicate whether each material, system or method listed is relevant to your study. If you are not sure if a list item applies to your research, read the appropriate section before selecting a response.

Materials & experimental systems

n/a	Involved in the study
☒	☐ Antibodies
☒	☐ Eukaryotic cell lines
☒	☐ Palaeontology
☒	☐ Animals and other organisms
☒	☐ Human research participants
☒	☐ Clinical data

Methods

n/a	Involved in the study
☒	☐ ChIP-seq
☒	☐ Flow cytometry
☒	☐ MRI-based neuroimaging
